# Complete genome sequence of a *Marinococcus* sp. PL1-022 isolated from the pink hypersaline Pearse Lakes, Rottnest Island, Western Australia

**DOI:** 10.1128/mra.00129-24

**Published:** 2024-07-05

**Authors:** Crystal E. Young, Hussain Alattas, Colin Scott, Daniel V. Murphy, Ravi Tiwari, Wayne G. Reeve

**Affiliations:** 1 Bioplastic Innovation Hub, Food Futures Institute, Murdoch University, Murdoch, Western Australia, Australia; 2 School of Medical, Molecular and Forensic Sciences, Murdoch University, Murdoch, Western Australia, Australia; 3 CSIRO Environment, Black Mountain Science and Innovation Park, Canberra, Australia; SUNY College of Environmental Science and Forestry, Syracuse, New York, USA

**Keywords:** extremophiles, halophiles, genomes

## Abstract

*Marinococcus* sp. PL1-022 was isolated from Pearse Lakes, Western Australia. The sequenced genome consists of a chromosome (3,140,198 bp; 48.2% GC) and two plasmids (58,083 bp and 19,399 bp; 41.4 and 50.7% GC-content, respectively). Isolation of *Marinococcus* sp. PL1-022 adds to the increasing repertoire of culturable extremophiles.

## ANNOUNCEMENT

*Marinococcus* are Gram-positive, motile, moderately halophilic cocci found in hypersaline environments (1). Rottnest Island (~30.4 km offshore of Perth, Western Australia) hosts athalassic soda-hypersaline lakes (2), including Pearse Lakes. We report the complete genome of a *Marinococcus* sp. PL1-022, isolated in May 2022, when the lakes had a pH of 8.0 ± 0.0 and salinity of 20.1 ± 1.4%

**Fig 1:**
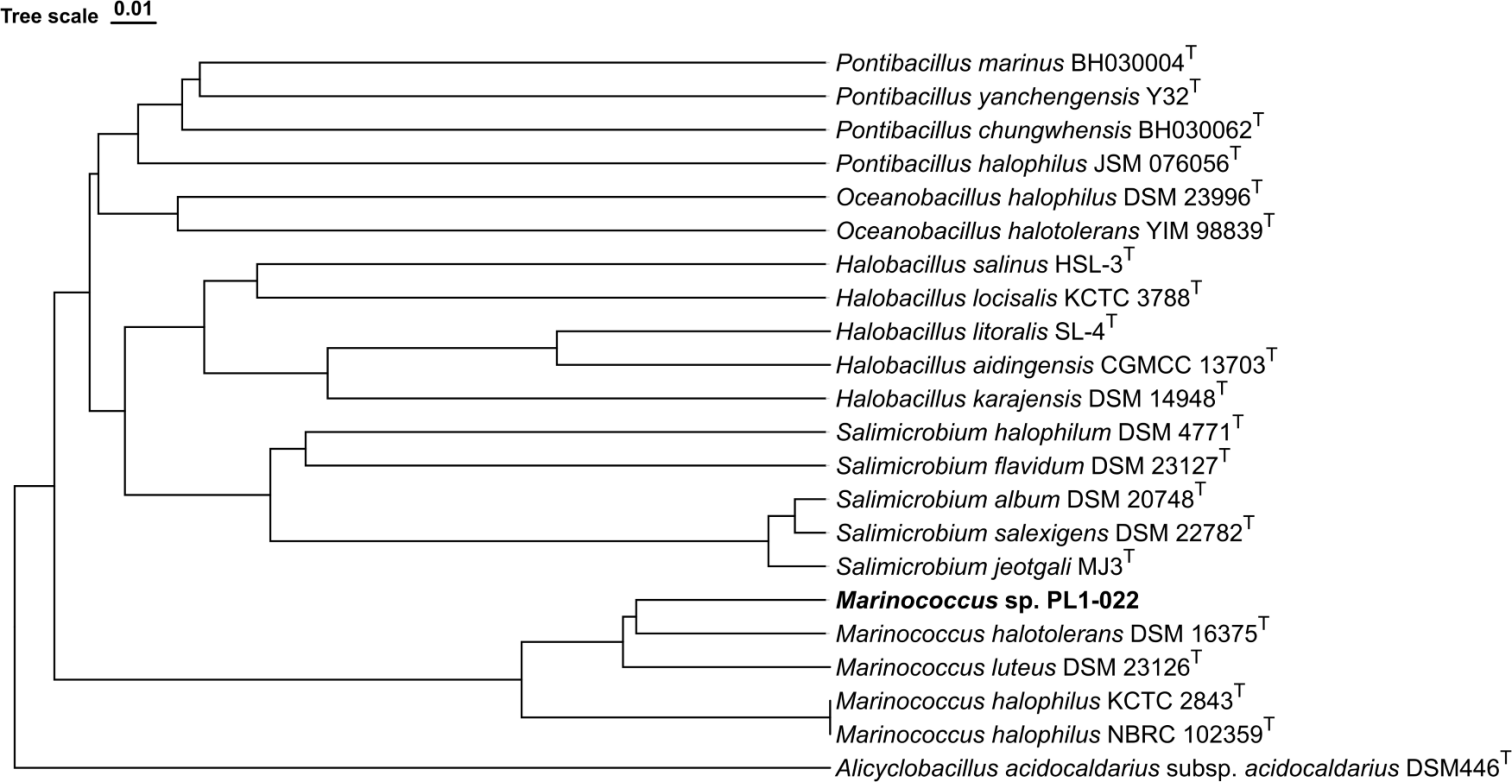
Dendro-unweighted pair group method with arithmetic mean (UPGMA) tree displaying the relatedness of *Marinococcus* sp. PL1-022 to related species based on ANIb values. ANIb values were generated using JSpeciesWS (10) and imported into DendroUPGMA (13), and the tree was constructed using a similarity matrix (Pearson’s correlation coefficient) within the algorithm (14). The tree was exported in Newick format and imported into tvBOT (https://www.tvBOT.html) (15). The superscript T indicates type strains and *Alicyclobacillus acidocaldarius* subsp. *acidocaldarius* DSM 446^T^ was used as an outgroup (16).

Water samples were collected (S 32°0′22.281″ E 115°30′44.484″) and stored at 4°C before cultivation. Water samples (1,500 mL) were centrifuged at 4,500 × *g* for 10 min and streaked from the cell pellet on adapted isolation medium containing (per liter): 10.0 g bacto-tryptone, 5.0 g bacto-yeast extract, 2.0 g KCl, 20.0 g MgSO_4_, 150.0 g NaCl, and 2.4 g HEPES (pH 7.8) (3). Single colonies were re-streaked until pure cultures were obtained and cryopreserved (15% glycerol, −80°C). Genomic DNA (gDNA) was isolated from stationary phase culture using the CTAB (2%) method (4) and sequenced using Oxford Nanopore Technology (ONT). The ONT library was prepared using the ONT ligation native barcoding gDNA library protocol (SQK-NBD114.24) (https://nanoporetech.com/protocols) with a FLO-PRO114M flow cell (R10.4.1) on the PromethION 2 platform. Guppy [v6.5.7 (5)] was used for base calling sequenced data with a read-pass-filter quality score cutoff value of nine and minimum length of 1,500 bp. 173,800 reads were generated (973,546,914 bp), providing an average coverage of 300× and a *N*_50_ value of 9,329 bp [NanoStat Ver. 1.6.0 (6)]. ONT Long reads were assembled using Flye [v2.9.2 (7)] (default parameters with nine iterations), providing a single circular chromosome (3,140,198 bp with 48.2% GC-content) and two circular plasmids (58,083 bp, 19,399 bp and 41.4%, 50.7% GC-content, respectively). A quantitative genome assembly assessment was performed using BUSCO [v5.4.6 (8)] and CheckM [v1.2.1 (9)] providing a completeness score of 99.1% and 91.8% (0.7% contamination), respectively. Average nucleotide identity analysis using BLAST (ANIb) showed a score below the cutoff value (>95%) with the closest relative (91.87%) *Marinococcus halotolerans* DSM 16375^T^ (GCA_000420725.1) (10 – 12) (Fig. 1).

Gene calling and annotation of the resulting complete genome was performed using the NCBI Prokaryotic Genome Annotation Pipeline (Table 1) [v6.6 (17)]. Isolation of *Marinococcus* sp. PL1-022 adds to the repertoire of culturable extremophiles and could be investigated to provide insight for microbial life at Pearse Lakes (18).

**TABLE 1:** General feature of the genome of *Marinococcus* sp. PL1-022 from prokaryotic genome annotation pipeline

	Data from:
Feature	GenBank Annotation
Total no. of genes	3,299
No. of protein coding sequences	3,198
No. of rRNA operons	5
5s	6
16s	5
23s	5
No. of tRNA genes	63
No. of other RNA genes	4
Locus Tag Prefix	SIC45_

## Data Availability

*Marinococcus* sp. PL1-022 whole-genome sequence (JAWXCX010000001) was deposited in GenBank (NCBI) under BioSample: SAMN38237612 within BioProject: PRJNA1039979. ONT long reads was deposited in SRA (SRR26963912).
